# Tylosin exposure reduces the susceptibility of *Salmonella* Typhimurium to florfenicol and tetracycline

**DOI:** 10.1186/s12917-020-2246-5

**Published:** 2020-01-28

**Authors:** Abraham Fikru Mechesso, Seung-Chun Park

**Affiliations:** Laboratory of Veterinary Pharmacokinetics and Pharmacodynamics, College of Veterinary Medicine, Kyungpook National University, Bukgu, Daegu, 41566 South Korea

**Keywords:** Florfenicol, *S*. Typhimurium, Susceptibility, Tetracycline, Tylosin

## Abstract

**Background:**

Antibiotics exposure has been implicated in the emergence of bacterial strains that are resistant to structurally related or unrelated antibiotics. Tylosin is a macrolide antibiotic that has been administered to treat respiratory pathogenic bacteria in swine. Thus, this study was undertaken to evaluate the impact of exposure to a constant (3 μg/mL) and decreasing concentrations of tylosin on the susceptibility of *Salmonella enterica* serovar Typhimurium to various antibiotics.

**Results:**

*S*. Typhimurium strains exposed to tylosin for 12 and 24 h in the in vitro dynamic model demonstrated at least an eight-fold increase in the minimum inhibitory concentrations (MICs) of florfenicol and tetracycline. Exposure to tylosin extended the lag-time of the growth curve and enhanced the generation of reactive oxygen species. Gene expression analysis demonstrated up-regulation of the *acrAB* and *tolC Salmonella* efflux pump genes and its global regulators (*marA* and *soxS*). Besides, the expression of *ompC* gene was down-regulated in tylosin exposed *S*. Typhimurium.

**Conclusion:**

Exposure to decreasing concentrations of tylosin could reduce the susceptibility of *S*. Typhimurium to florfenicol and tetracycline.

## Background

Multidrug-resistant strains of *Salmonella enterica* serovar Typhimurium have become a major concern in both veterinary and human medicine. It is one of the main causes of food-borne gastroenteritis in humans [1]. Swine are asymptomatic carriers of *S*. Typhimurium and serve as reservoirs of infection [2]. Although infection is usually associated with self-limiting gastroenteritis, septicaemic cases associated with severe clinical signs and sudden death have been reported in swine [3].

Tylosin is mainly used to control respiratory infections caused by Gram-positive bacteria and Mycoplasma species in farm animals including swine. Although it is prohibited in Europe and some Asian countries, it has been used as a livestock feed additive and antibiotic growth promoter in other parts of the world [4, 5]. Sub-inhibitory concentrations of antibiotics have been reported to induce the emergence of antibiotic resistance [6]. Studies have shown the emergence of antibiotic-resistant bacteria following prior exposure to sub-therapeutic doses of structurally related or unrelated antibiotic (s). Aarestrup and Carstensen [7] have demonstrated that the use of tylosin as a growth promoter in pigs contributed to the emergence of resistant enterococci and *Staphylococcus hyicus*. A study by Fung-Tomc et al. [8] revealed that pre-exposure of methicillin-resistant *Staphylococcus aureus* and *Pseudomonas aeruginosa* to sub-inhibitory concentrations of ciprofloxacin resulted in resistance to structurally unrelated antibiotics, including tetracycline and gentamicin. In addition, exposure to sub-inhibitory concentrations of kanamycin and streptomycin is reported to induce resistance to structurally unrelated antibiotics in *Streptococcus pneumoniae* and *Escherichia coli* [9, 10].

Despite the widespread uses of tylosin in the livestock industry, especially swine, there is no report on the impacts of tylosin exposure on the antibiotic susceptibility profiles of Gram-negative bacteria including *S*. Typhimurium. In this study, we attempted to examine the impacts of exposure to a constant (3 μg/mL) and decreasing concentrations of tylosin on the susceptibilities of *S*. Typhimurium strains to selected antibiotics that are commonly used in veterinary medicine. We have also determined the tylosin-induced changes in the gene expression of *S*. Typhimurium efflux pump and outer membrane porins.

## Results

### Effects of tylosin exposure on the susceptibility of *S*. Typhimurium

The MICs of the selected antibiotics against *S*. Typhimurium strains before and after exposure to tylosin in the dynamic model (taken after 12 and 24 h of incubation) and in the presence (EI) and absence (WEI) of Phe-Arg-β-naphthylamide (an efflux pump inhibitor) is summarized in Table 1. Non-exposed strains of *S*. Typhimurium LVPP-STI2 and LVPP-STI15 were resistant to sulfamethoxazole and streptomycin while trimethoprim and tetracycline resistance was observed only in *S*. Typhimurium LVPP-STI15. Tylosin exposed *S*. Typhimurium ATCC 14028 and LVPP-STI2 demonstrated higher MICs of florfenicol and tetracycline (by at least 8-fold) compared to the non-exposed counterparts. However, exposure did not change the MICs of florfenicol and tetracycline in *S*. Typhimurium LVPP-STI15. The tylosin-induced increase in the MICs of florfenicol and tetracycline were reduced to various levels in the presence of Phe-Arg-β-naphthylamide. In contrast, exposure of *S*. Typhimurium strains to tylosin in the dynamic model did not cause a significant change in the MICs of marbofloxacin, streptomycin, sulfamethoxazole and trimethoprim. Similarly, exposure to tylosin for 1, 2, 4, and 8 h in the dynamic model and at all-time points in the static model demonstrated slight or no change in the MICs of the tested antibiotics (Additional file 1). Therefore, *S*. Typhimurium strains that were exposed to tylosin for 12 h in the dynamic model were considered for subsequent experiments.

**Table 1:** The MICs (μg/mL) of the selected antibiotics against *S*. Typhimurium strains before and after exposure to tylosin (taken after 12 and 24 h of incubation) in the in vitro dynamic model and in the presence and absence of an efflux pump inhibitor

Antibiotics	*S*. Typhimurium (ATCC-14028)				*S*. Typhimurium (LVPP-STI2)				*S*. Typhimurium (LVPP-STI15)			
	Pre		Post		Pre		Post		Pre		Post	
	WEI	EI	WEI	EI	WEI	EI	WEI	EI	WEI	EI	WEI	EI
TET	2	2	128	8	1	1	128	4	256	64	512	128
MBF	0.03	0.03	0.06	0.06	0.03	0.03	0.06	0.03	0.5	0.13	2	0.26
FFL	4	4	32	8	4	2	32	8	2	1	8	2
TMP	0.04	0.04	0.04	0.04	0.04	0.04	0.16	0.16	> 256	> 256	> 256	> 256
STR	32	32	512	64	1024	512	1024	1024	1024	1024	> 1024	> 1024
SMT	64	64	64	64	> 1024	1024	> 1024	> 1024	> 1024	> 1024	> 1024	> 1024
TYL	1024	512	1024	512	1024	1024	1024	1024	1024	1024	1024	1024

SMX = sulfamethoxazole, TET = tetracycline, FFL = florfenicol, TMP = trimethoprim, MBF = marbofloxacin, STR = streptomycin, and TYL = tylosin, WEI = without the efflux pump inhibitor, EI = together with the efflux pump inhibitor (Phe-Arg-β-naphthylamide, 40 μg/mL). The MIC values were similar for both time points (12 vs 24 h)

### Growth curves of *S*. Typhimurium

**Fig. 1:**
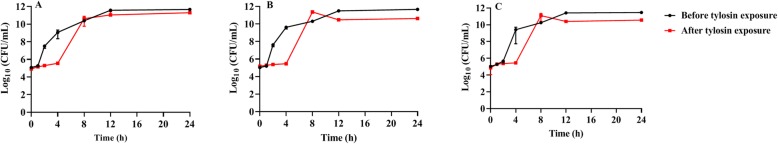
The growth curves of *S*. Typhimurium ATCC14028 (**a**), LVPP-STI2 (**b**) and LVPP-STI15 (**c**) before and after exposure to decreasing concentration of tylosin (initial concentration of 3 μg/mL) in the in vitro dynamic assay. There was not a time-dependent difference (12th vs 24th h) in the growth curve of *S*. Typhimurium strains. Therefore, representative figures for both time points are displayed here

Figure 1 shows the growth curves of *S*. Typhimurium strains before and after exposure to tylosin in the dynamic model. The duration of lag-time in tylosin exposed *S*. Typhimurium strains ATCC 14028 and LVPP-STI2 were longer (~ 4.5 h) than the non-exposed counterparts (~ 1.5–2 h). Treatment with tylosin extended the lag-time of *S*. Typhimurium LVPP-STI15 only by approximately 1.5 h. However, once the strains reached stationary phase (~ 10 h); the differences were negligible.

### Tylosin-induced free radical generation in *S*. Typhimurium

**Fig. 2:**
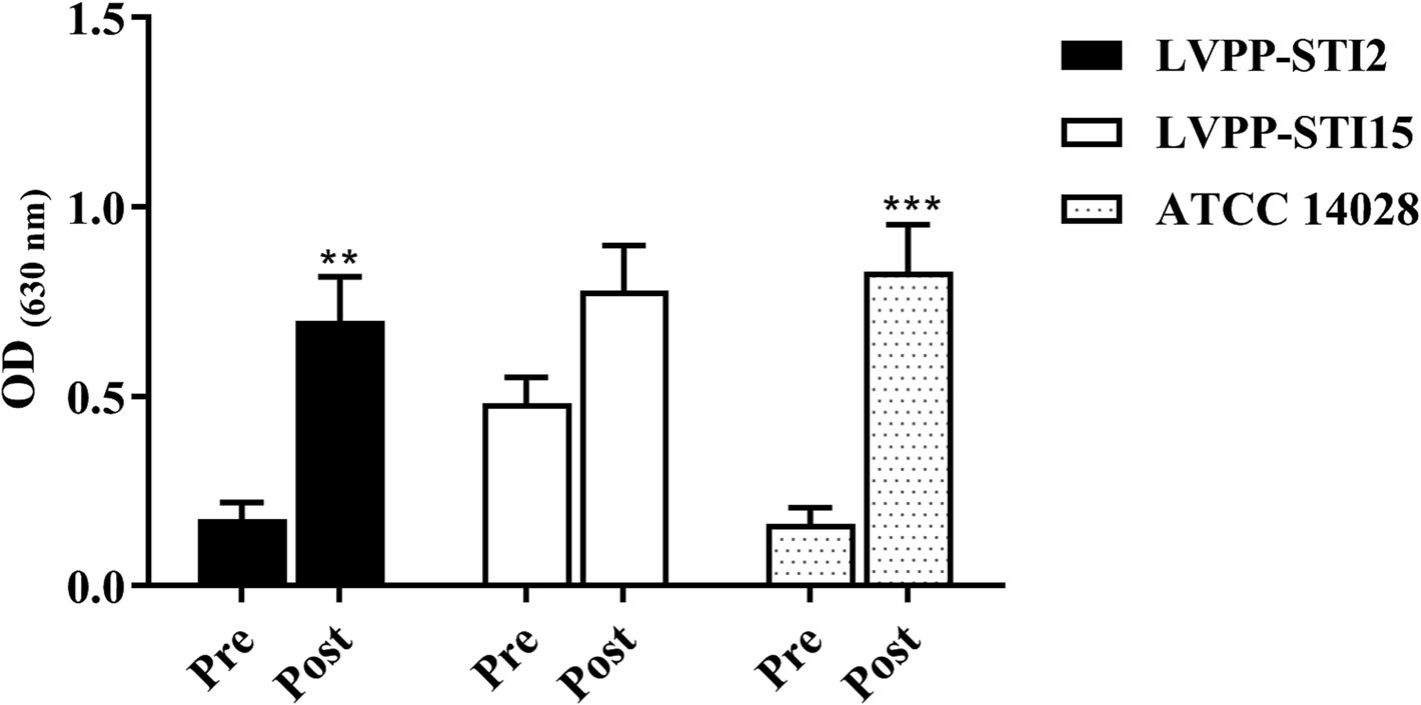
Generation of free radicals (measured in terms of optical density) in *S*. Typhimurium before and after exposure to decreasing concentration of tylosin (initial concentration of 3 μg/mL) in the in vitro dynamic assay. Data presented as mean ± SD. **P < 0.01 compared to the non-exposed *S*. Typhimurium

In the Nitro blue tetrazolium (NBT) assay, the extent of free radicals generated in tylosin exposed *S*. Typhimurium ATCC 14028 and LVPP-STI 2 were significantly higher (*P < 0.01*) compared to its counterparts before exposure (Fig. 2). However, tylosin did not cause a significant change in free radical generation in *S*. Typhimurium LVPP-STI15.

**Fig. 3:**
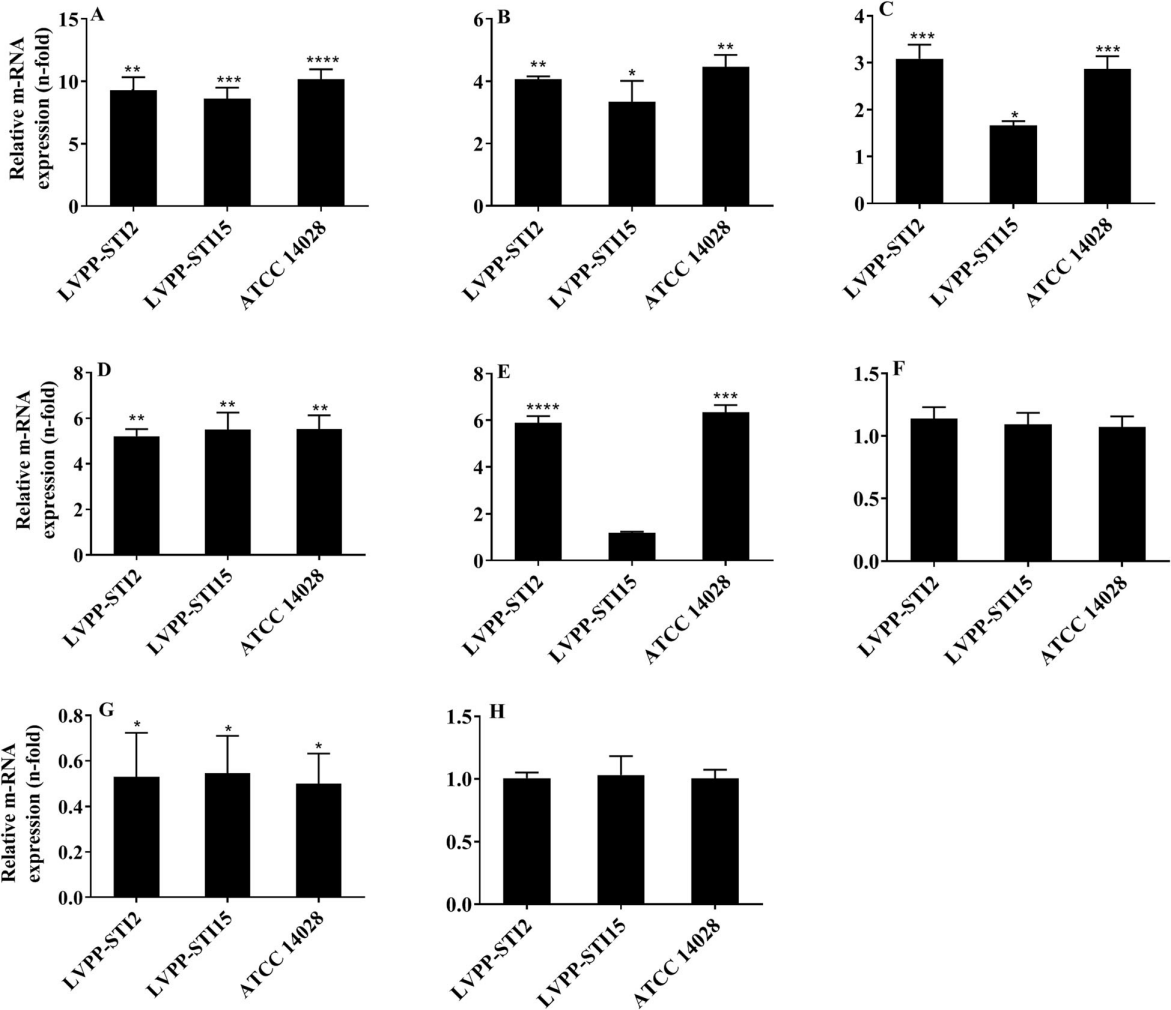
Effects of 12 h exposure to decreasing concentration of tylosin (initial concentration of 3 μg/mL) on the gene expression of *S*. Typhimurium efflux pump: *acrA* (**a**) *acrB* (**b**) *tolC* (**c**), and genes encoding their global regulators *marA* (**d**) *soxS* (**e**) *ramA* (**f**). The gene expression value for the non-exposed *S*. Typhimurium is 1. The gene expression of outer membrane proteins: *ompC* (**a**) and *ompF* (**a**) are displayed in Fig. **g** and **h**, respectively. Bar graphs indicate the mean ± SD of three independent experiments. *P < 0.05; ** *P < 0.01*; *** *P < 0.001* compared to the non-exposed *S*. Typhimurium

### Effects of tylosin treatment on *S*. Typhimurium efflux-pump and outer membrane proteins

Figure 3(a-f) shows the role of tylosin on the gene expression of *acrA*, *acrB*, and *tolC*, and the *marA*, *soxS*, and *ramA* genes encoding their global regulators. Treatment with tylosin significantly increased the gene expression of *acrA*, *acrB*, and *tolC* by 8.6-to-10.2 fold (*P < 0.001*), 3.3-to-4.5 fold (*P < 0.05*), and 1.7-to-3.1 fold (*P < 0.05*), respectively relative to the non-exposed counterparts. Similarly, tylosin treated *S*. Typhimurium strains demonstrated 5.2-to 5.5-fold (*P < 0.01*) and 5.9-to 6.3-fold (*P < 0.001*) increases in expression for the *marA* and *soxS* genes, respectively in *S*. Typhimurium strains ATCC 14028 and LVPP-STI2 compared to the non-exposed counterparts. However, the changes in tylosin-induced expression of *ramA* (in all strains) and *soxS* genes in *S*. Typhimurium LVPP-STI15 were not significant (*P < 0.05*). Tylosin exposure down-regulated expression of the *ompC* gene by 44.5–50.2% (*P < 0.05*), while the change in *ompF* gene expression was insignificant (*P > 0.05*) (Fig. 3g and h)

## Discussion

Only after a few years of initiation of antimicrobials use in humans, the selective pressure was identified as a driving force behind the emergence of resistant bacteria. Resistance is either genetically encoded which is then inherited by subsequent progeny of the resistant pathogens and in some cases could be transferred horizontally even to distantly related bacteria [11]. Acquisition of plasmid-mediated resistant genes is the principal mechanism for antimicrobial resistance in *Salmonella*. However, spontaneous gene mutation does contribute to the emergence of resistant *Salmonella* strains especially to quinolones [12, 13].

The selection of drug-resistant mutants at high antibiotic concentrations is a well-known fact. Exposure to high antibiotic concentrations exceeding the MIC interferes with the survival of susceptible bacteria, hence susceptible cells will no longer grow and are therefore outcompeted by resistant ones [14]. However, the role of exposure to antibiotics below their MIC has only recently gained importance in this context. Most importantly, the effects of sub-inhibitory concentrations of antibiotics in the evolution of resistant bacterial strains, especially to structurally unrelated antibiotics are less delineated. Exposure to sub-therapeutic doses of antibiotics may cause phenotypic and genetic variability and act as selectors of resistance [15]. The selection of antibiotic resistance at sub-inhibitory concentrations differs from that of the lethal drug concentration in various aspects. Selection at sub-inhibitory concentrations is progressive and is strongly associated with mutations that have a low fitness cost. In addition, it ensures greater mutational space and favors the accumulation of multiple small step mutations [15–17].

Tylosin has limited activity against Gram-negative bacteria, especially those belonging to the *Enterobacteriaceae*, because of reduced penetration of the outer membrane [4]. In this study, the MIC of tylosin against the tested *S*. Typhimurium strains was 1024 μg/mL. In both the static and dynamic assays, *Salmonella* strains were exposed to 3 μg/mL of tylosin which is sub-lethal and had no impact on bacterial survival. It should also be taken into account that the concentration of tylosin in the dynamic model decreases through time from the initial 3 μg/mL because of the infusion of fresh medium and dilution of tylosin from the central compartment.

Previous studies have demonstrated that exposure to sub-MICs of antibiotics could lead to the emergence of multidrug-resistant strains through free radical (reactive oxygen species, ROS) induced mutagenesis [18]. In this study, we have observed that the levels of free radicals generated in tylosin exposed *S*. Typhimurium were significantly higher than the level in the non-exposed counterparts. Consistent with our findings, Kohanski et al. [19] demonstrated the association between the reduced susceptibility, antibiotics induce mutagenesis, and ROS generation in various bacteria, including *E.coli* following exposure to sub-lethal doses of antibiotics. Besides, a recent study by Li et al. [20] illustrated the generation of ROS and the subsequent emergence of vancomycin-resistant *Staphylococcus aureus* after treatment with sub-MIC levels of vancomycin.

Membrane permeability is one of the critical factors in regulating the antibiotic susceptibility of *Enterobacteriaceae* such as *S*. Typhimurium*.* Alteration of the bacterial envelope through the reduction of porin production or increased expression of efflux pump systems contributed significantly to the emergence of resistant bacteria [21]. Tetracyclines enter into *S*. Typhimurium via the outer membrane porins, OmpC or OmpF [22]. Therefore the interaction between the antibiotics and the bacterial target sites is dependent on the level of expression of these porins. This study demonstrated that the expression of *ompC* gene was significantly (*P* < 0.05) down-regulated following exposure to tylosin. Down-regulation of these porins reduces the diffusion of tetracyclines into the cytoplasm target sites and ultimately results in 6-to-18 fold increase in the MIC as reported in other Gram-negative bacteria. Besides, bacteria that acquire such type of resistance through suppression of porin could also become resistant to other antibiotics [23]. However, tylosin exposure did not produce a significant change in the expression of *ompF* gene (*P* > 0.05). The selective effect of tylosin on *ompC* gene expression is not surprising because previous studies in Gram-negative bacteria confirmed that the expression of these genes may vary depending upon the osmolarity of culture medium and type of chemical exposed [24, 25].

Activation of efflux pumps (*acrAB-tolC*) and reducing intracellular concentrations of tetracycline and florfenicol is the most familiar resistance mechanisms of Gram-negative bacteria [26]. The expression of *acrA*, *acrB*, and *tolC* was up-regulated to various extents in tylosin exposed *S*. Typhimurium strains with high MICs to florfenicol and tetracycline compared to the non-exposed counterparts. The decisive roles played by the AcrAB-TolC efflux system in acquiring multidrug resistance has also been explained by previous studies [27, 28]. Furthermore, the expression of the global regulators of *acrA*, *acrB*, and *tolC* such as *marA*, and *soxS* were significantly up-regulated in tylosin-exposed *S*. Typhimurium strains compared to the non-exposed counterparts. Studies confirmed that these global regulators are critical for the activation of the transcription of *acrA*, *acrB*, and *tolC* [29, 30]. This substantiates the role played by tylosin in activating the *acrAB-tolC* efflux system and its global regulators, which subsequently increases the MICs of florfenicol and tetracycline against *S*. Typhimurium. Therefore, reduced influx via altered porin phenotypes and simultaneous increased production of the AcrAB-tolC efflux pump and its global regulators could contribute to the higher MICs of florfenicol and tetracycline in *S*. Typhimurium strains pre-treated with tylosin.

Exposure to tylosin extended the lag-time of *S*. Typhimurium strains by approximately 2–2.5 h. Most environmental strains of bacteria become resistant to the action of tetracycline by extending the lag-time [31]. Besides, a study by Fridman et al. [32] demonstrated that change in the lag-time is one of the most essential changes made by the bacterium to develop tolerance in response to antibiotic stress. Extension of lag-time is associated with the survival of bacteria in the presence of antibiotics beyond the MICs and subsequently facilitates the evolution of resistant bacteria. Moreover, the extension of the lag phase allows the bacteria to survive and re-grow when optimal conditions arise. Overall, the differences in bacterial strains used in this study could contribute to the variations in the tylosin-induced changes in the MICs, free radical generation and gene expression in *S*. Typhimurium strains. Tylosin exposed *S*. Typhimurium strains exhibited similar MICs and gene expression patterns of efflux pumps following passage for multiple generations, indicating phenotypic and gene expression stability of the strains.

## Conclusions

Exposure of *S*. Typhimurium to decreasing concentration of tylosin increases the MICs of florfenicol and tetracycline mainly through extending the lag phase of bacterial growth, enhancing ROS generation, and activating the *acrAB-tolC* efflux system. Future work will aim at investigating the molecular changes associated with the increased MICs florfenicol and tetracycline in tylosin-exposed *S*. Typhimurium.

## Methods

### Chemicals and reagents

The antibiotics, chemicals and reagents used in this study were purchased from Sigma (St. Louis, MO, USA) unless indicated. Stock solutions of florfenicol (FFL), sulfamethoxazole (SMX), tetracycline (TET) and trimethoprim (TMP) were prepared in dimethyl sulfoxide (DMSO). Marbofloxacin (MBF) and streptomycin (STR) were prepared in sterile distilled water. Besides, a stock solution of tylosin (TYL) was made in 50% ethanol. The stock preparations were diluted in sterile distilled water or appropriate broth medium. The concentrations of DMSO and ethanol in the final diluents never exceeded 0.1% (v/v).

### *S*. Typhimurium strains and culture conditions

*S*. Typhimurium strains (LVPP-STI2 and LVPP-STI15) isolated from swine [33] and *S*. Typhimurium ATCC14028 were cultured in Luria-Bertani (LB) - agar (Difco, MD, USA) at 37 °C. Before assays, the bacteria were grown overnight in LB-broth at 37 °C in a shaking incubator. Our previous study revealed that the parent *S*. Typhimurium LVPP-STI15 possesses a single *gyrA* mutation resulting in amino acid substitution, Asp87Tyr, whereas no mutations were identified in the parent *S.* Typhimurium LVPP-STI2 [33]. In addition, our recent studies confirmed the invasive, quorum sensing and virulence potentials of these strains [34, 35].

### In vitro static tylosin therapy

*S*. Typhimurium strains were cultured in 10 mL Mueller–Hinton broth (MHB) containing 3 μg/mL of tylosin and incubated at 37 °C in a shaking incubator. The concentration of tylosin was determined based on the average results of the maximum plasma concentration of tylosin in swine from previous studies [36–38]. Twenty microliters of samples were taken at 0, 1, 2, 4, 6, 8, 12 and 24 h from the time of incubation and cultured in LB-agar plates. The experiment was conducted in duplicate in three separate experiments.

### Tylosin therapy using the in vitro dynamic model

A previously described in vitro dynamic model which contains a dilution compartment containing fresh Muller-Hinton broth II (MHB II), a central compartment with either a bacterial culture alone (control growth experiment) or a bacterial culture in medium containing tylosin (killing-regrowth experiments), and an elimination compartment containing waste broth and bacteria was used to expose *S*. Typhimurium strains to tylosin [39]. A magnetic stirrer was placed in both flasks in the central compartment. Peristaltic pumps (Masterflex, Cole-Parmer, USA) circulated in one direction, from the dilution compartment to the central compartment and from the central compartment to the elimination compartment, at a flow rate of 8.1 mL/h that corresponds to average half-life of tylosin in swine [36, 37]. The system was placed in an incubator at 37 °C. Six hundred microliter of an overnight culture of *S*. Typhimurium was inoculated into the central compartment. After 2 h of incubation, the bacterial cultures reached 10^8^ CFU/mL (6 × 10^9^ CFU per 60 mL central compartment). Tylosin (3 μg/mL) was injected into one of the units in the central compartment. Then, 20 μL aliquot samples were collected from each compartment in the central unit at 0, 1, 2. 4, 8, 12, and 24 h. Samples were cultured on LB-agar plates and incubated at 37 °C for 24 h. The experiment was conducted in triplicate for each isolate.

### Determination of MIC in the presence and absence of an efflux pump inhibitor

The minimum inhibitory concentration (MICs) of florfenicol, marbofloxacin, streptomycin, sulfamethoxazole, tetracycline and trimethoprim against *S*. Typhimurium strains were determined before and after (at 0, 1, 2, 4, 8, 12 and 24 h post-incubation) exposure to the static and dynamic tylosin treatment. The MICs were determined in the presence and absence of an efflux pump inhibitor (Phe-Arg-β-naphthylamide, 40 μg/mL) using the broth microdilution method with an inoculum of approximately 10^5^ CFU/mL. Four colonies isolated from tylosin exposure/time point were assayed and this was repeated for three experiments i.e. a total of at least 12 colonies assayed for a single exposure/time point. The lowest concentrations of antibiotics inhibiting visible bacterial growth after incubation at 37 °C for 18–24 h were considered as MICs. Clinical breakpoints for tetracycline, florfenicol and sulfamethoxazole were: TET ≥ 32 μg/mL, FFL ≥ 16 μg/mL and SMT ≥ 512 μg/mL. Since CLSI breakpoints were not available for marbofloxacin, streptomycin and trimethoprim, the following breakpoints were used: MBF ≥ 1 μg/mL (ciprofloxacin), STR ≥ 64 μg/mL and TMP ≥ 2 μg/mL [40–42]. In addition, tylosin is known to be inactive against Gram-negative bacteria and its MIC in *Salmonella* is ≥1 mg/mL [34].

### Growth curves

Based on the results of the MIC assay, the growth curves of *S*. Typhimurium strains were determined before and after exposure to tylosin for 12 h in the dynamic model. Briefly, *S*. Typhimurium (10^5^ CFU/mL) was incubated at 37 °C in MH-broth in a shaking incubator. After 0, 1, 2, 4, 8, 12 and 24 h from the time of incubation, 100 μL of the suspension was removed and diluted serially (10-fold). Then, 20 μL of the dilutions were spread plated on LB-agar plates and the CFUs were determined following incubation at 37 °C for 24 h.

### Estimation of free radicals

NBT assay was performed to determine the amount of free radicals generated by *S*. Typhimurium before and after (12 h) exposure to tylosin using a minor modification of a previous method [11]. The quantity of formazan crystals that are produced from water-soluble tetrazolium salt within each bacterial cell is directly proportional to the production of free radicals [43]. Briefly, 4 h cultures of *S*. Typhimurium were diluted to 10^6^ CFU/mL in LB-broth. Bacteria were centrifuged at 500 x*g* for 10 min and washed twice in 1× PBS. The pellet was then suspended in 1× PBS (200 μL). Freshly prepared NBT (0.01%) solution was added and incubated at 37 °C for 1 h. Then, it was washed again with 1× PBS and centrifuged at 500 x*g* for 10 min. The blue, water-insoluble intracellular formazan crystals were dissolved in 60 μL potassium hydroxide solution (2 M, DMSO). Thereafter, the preformed bacterial superoxide anions were quantified using a microplate reader at 630 nm (VersaMax, Molecular Devices, CA, USA). The experiment was conducted in duplicate in three different experiments for each strain.

### QRT-PCR analysis of *Salmonella* efflux pump, the efflux pump global regulators, and outer membrane porins

Quantitative reverse transcription-PCR (qRT-PCR) analysis was performed to determine the impacts of tylosin exposure (12 h) on the gene expression of *acrA*, *acrB*, and *tolC* (encoding the *S*. Typhimurium AcrAB-TolC efflux pump), *marA*, *soxS,* and *ramA* (encoding their global regulators), and *ompC* and *ompF* (encoding outer membrane porins). Total RNA was extracted using TRIzol (Ambion Life Technologies, Carlsbad, CA, USA) and qRT-PCR was conducted similarly to O’Regan et al. [29]. Gene expression levels of *acrA*, *acrB*, and *tolC*, *marA*, *soxS*, *ramA*, *ompC* and *ompF* were determined by CFX96 Touch™ real-time PCR detection system (174 Biorad, USA) using IQ™ SYBR® Green Supermix (Biorad, Singapore). The reaction conditions include denaturation at 94 °C for 3 min, followed by 35 cycles of amplification. Each cycle of amplification consists of 1 min at 94 °C, 20–60 s at the appropriate annealing temperature (Additional file 2), and 1 min at 72 °C. The final extension step was at 72 °C for 10 min. The housekeeping gene *rrsG* was used to normalize gene expression (2^-ΔΔCT^). The primers used in this experiment are listed in Additional file 2.

### Data analysis

Data were analyzed using GraphPad Prism 6 (GraphPad Software, Inc., San Diego, CA, USA). One-way analyses of variance (ANOVA) followed by Tukey’s HSD test were conducted to compare the mean values among treatment groups. *p* < 0.05 was considered statistically significant.

## Supplementary information

**Additional file 1: Table S1.** The MICs (μg/mL) of selected antibiotics against *S*. Typhimurium strains before and after exposure to tylosin (taken after 1 and 2 h of incubation in the in vitro dynamic model and at all tylosin exposure/time points in the static model) in the presence and absence of an efflux pump inhibitor. **Table S2.** The MICs (μg/mL) of selected antibiotics against *S*. Typhimurium strains before and after exposure to tylosin (taken after 4 and 8 h of incubation) in the in vitro dynamic model and in the presence and absence of an efflux pump inhibitor.

**Additional file 2.** Lists of primer sequences.

## Data Availability

All data generated or analyzed during this study are included in this published article.

## Abbreviations

ANOVA: Analysis of variance
CFU: Colony-forming unit
LB: Luria–Bertani
MHB: Mueller–Hinton broth
MIC: Minimum inhibitory concentration
qRT–PCR: quantitative real-time reverse transcription PCR
ROS: Reactive oxygen species
