# Supplementary material for: Tylosin exposure reduces the susceptibility of Salmonella Typhimurium to florfenicol and tetracycline
Source: BMC Vet Res. 2020 Jan 28;16:22. doi: 10.1186/s12917-020-2246-5 (PMC6986020; doi:10.1186/s12917-020-2246-5)
Supplement: Supplementary file 1 — Additional file 1: Table S1. The MICs (μg/mL) of selected antibiotics against S. Typhimurium strains before and after exposure to tylosin (taken after 1 and 2 h of incubation in the in vitro dynamic model and at all tylosin exposure/time points in the static model) in the presence and absence of an efflux pump inhibitor. Table S2. The MICs (μg/mL) of selected antibiotics against S. Typhimurium strains before and after exposure to tylosin (taken after 4 and 8 h of incubation) in the in vitro dynamic model and in the presence and absence of an efflux pump inhibitor. [file 12917_2020_2246_MOESM1_ESM.docx]

**Table S1**. The MICs (µg/mL) of selected antibiotics against *S*. Typhimurium strains before and after exposure to tylosin (taken after 1 and 2 h of incubation in the *in vitro* dynamic model and at all tylosin exposure/time points in the static model) in the presence and absence of an efflux pump inhibitor.

| Antibiotics | *S*. Typhimurium (ATCC-14028) | | | | *S*. Typhimurium (LVPP-STI2) | | | | *S*. Typhimurium (LVPP-STI15) | | | |
| --- | --- | --- | --- | --- | --- | --- | --- | --- | --- | --- | --- | --- |
|  | Pre | | Post | | Pre | | Post | | Pre | | Post | |
|  | WEI | EI | WEI | EI | WEI | EI | WEI | EI | WEI | EI | WEI | EI |
| TET | 2 | 2 | 2 | 2 | 1 | 1 | 1 | 1 | 256 | 64 | 256 | 64 |
| MBF | 0.03 | 0.03 | 0.03 | 0.03 | 0.03 | 0.03 | 0.06 | 0.03 | 0.5 | 0.13 | 0.5 | 0.13 |
| FFL | 4 | 4 | 4 | 4 | 4 | 2 | 4 | 2 | 2 | 1 | 2 | 2 |
| TMP | 0.04 | 0.04 | 0.04 | 0.04 | 0.04 | 0.04 | 0.04 | 0.04 | > 256 | > 256 | > 256 | > 256 |
| STR | 32 | 32 | 64 | 32 | 1024 | 512 | 1024 | 512 | 1024 | 1024 | > 1024 | > 1024 |
| SMT | 64 | 64 | 64 | 64 | > 1024 | 1024 | > 1024 | 1024 | > 1024 | > 1024 | > 1024 | > 1024 |
| TYL | 1024 | 512 | 1024 | 512 | 1024 | 1024 | 1024 | 1024 | 1024 | 1024 | 1024 | 1024 |

SMX= sulfamethoxazole, TET=tetracycline, FFL=florfenicol, TMP=trimethoprim, MBF= marbofloxacin, STR =streptomycin, and TYL= tylosin, WEI= without the efflux pump inhibitor, EI= together with the efflux pump inhibitor (Phe-Arg-β-naphthlamide, 40 µg/mL). The MIC values were similar for all tylosin exposure/time points.

| Antibiotics | *S*. Typhimurium (ATCC-14028) | | | | *S*. Typhimurium (LVPP-STI2) | | | | *S*. Typhimurium (LVPP-STI15) | | | |
| --- | --- | --- | --- | --- | --- | --- | --- | --- | --- | --- | --- | --- |
|  | Pre | | Post | | Pre | | Post | | Pre | | Post | |
|  | WEI | EI | WEI | EI | WEI | EI | WEI | EI | WEI | EI | WEI | EI |
| TET | 2 | 2 | 4 | 2 | 1 | 1 | 4 | 4 | 256 | 64 | 256 | 64 |
| MBF | 0.03 | 0.03 | 0.03 | 0.03 | 0.03 | 0.03 | 0.06 | 0.03 | 0.5 | 0.13 | 0.5 | 0.13 |
| FFL | 4 | 4 | 8 | 4 | 4 | 2 | 8 | 8 | 2 | 1 | 2 | 2 |
| TMP | 0.04 | 0.04 | 0.04 | 0.04 | 0.04 | 0.04 | 0.04 | 0.04 | > 256 | > 256 | > 256 | > 256 |
| STR | 32 | 32 | 128 | 64 | 1024 | 512 | 1024 | 1024 | 1024 | 1024 | > 1024 | > 1024 |
| SMT | 64 | 64 | 64 | 64 | > 1024 | 1024 | > 1024 | > 1024 | > 1024 | > 1024 | > 1024 | > 1024 |
| TYL | 1024 | 512 | 1024 | 512 | 1024 | 1024 | 1024 | 1024 | 1024 | 1024 | 1024 | 1024 |

**Table S2**. The MICs (µg/mL) of selected antibiotics against *S*. Typhimurium strains before and after exposure to tylosin (taken after 4 and 8 h of incubation) in the *in vitro* dynamic model and in the presence and absence of an efflux pump inhibitor.

SMX= sulfamethoxazole, TET=tetracycline, FFL=florfenicol, TMP=trimethoprim, MBF= marbofloxacin, STR =streptomycin, and TYL= tylosin, WEI= without the efflux pump inhibitor, EI= together with the efflux pump inhibitor (Phe-Arg-β-naphthylamide, 40 µg/mL). The MIC values were similar for both time points (4 vs 8 h).
