# Supplementary material for: Tylosin exposure reduces the susceptibility of Salmonella Typhimurium to florfenicol and tetracycline
Source: BMC Vet Res. 2020 Jan 28;16:22. doi: 10.1186/s12917-020-2246-5 (PMC6986020; doi:10.1186/s12917-020-2246-5)
Supplement: Supplementary file 2 — Additional file 2. Lists of primer sequences. [file 12917_2020_2246_MOESM2_ESM.docx]

| Target gene | Primer sequence | Annealing temperature in °C (time in sec.) |
| --- | --- | --- |
| *acrA* | F-5′- AAAACGGCAAAGCGAAGGT-3′ | 59 (20 s) |
|  | R-5′- GTACCGGACTGCGGGAATT -3′ |  |
| *acrB* | F-5′- TGAAAAAAATGGAACCGTTCTTC-3′ | 59 (20 s) |
|  | R-5′- CGAACGGCGTGGTGTCA3′ |  |
| *marA* | F-5′- ATCCGCAGCCGTAAAATGAC -3′ | 59 (1 min) |
|  | R-5′- TGGTTCAGCGGCAGCATATA -3′ |  |
| *ompC* | F-5′- CGCGGATCCATGAAAGTTAAAGTACTGTCCCTCCTG-3′ | 59 (45 s) |
|  | R-5′- CCCAAGCTTCCCGCAGGCCCTTTAGCAACAT -3′ |  |
| *ompF* | F-5′-CCTGGCAGCGGTGATCC-3′ | 58 (20 s) |
|  | R-5′-AAATTTCTGCTGCGTTTGCG-3′ |  |
| *ram A* | F-5′-CGTCATGCGGGGTATTCCAAGTG-3′ | 59 (1 min) |
|  | R-5′-CGCGCCGCCAGTTTTAGC-3′ |  |
| *rrsG* | F-5′-GTTACCCGCAGAAGAAGCAC-3′ | 59 (20 s) |
|  | R-5′- CACATCCGACTTGACAGACC 3′ |  |
| *soxS* | F-5′- AAATCGGGCTACTCCAAGTG-3′ | 59 (1 min) |
|  | R-5′- CTACAGGCGGTGACGGTAAT -3′ |  |
| *tolC* | F-5′-GCCCGTGCGCAATATGAT-3′ | 59 (20 s) |
|  | R-5′-CCGCGTTATCCAGGTTGTTG-3′ |  |

**Additional file 2**. Lists of primer sequences
